# A Display in Domestic Economy

**Published:** 1918-07-06

**Authors:** 


### A Display in Domestic Economy.

"ONCE in a blue moon, and not so often in war-time," London Domestic Economy Centres invite parents and friends to an Open Day. One such took place in the first week in June at Southampton Street Centre, Camberwell, when proud little cooks stood by tables covered with excellent war-time food of every variety, and—most important point—very daintily dished up. Why not, if and when you have made lentil cutlets, finish each with a frill and stand them in a row on a bed of mashed potato, with a little heap of green peas decorating on each side the surrounding gravy? If the whole of this family dish can be sold at 11½d. to allow for use of fire, it may as well look tempting. In the laundry, other girls showed that they had so skilfully mangled table linen as to make an iron unnecessary. They had not only washed and got up every ordinary kind of garment, but had entirely renovated a man's serge trousers and a woman's serge skirt, and had cleaned straw hats, re-trimmed them with folds of soft muslin dyed with coloured inks to delicate—and even—shades of mauve and blue and pink. Up in the bedroom of the "household work" flat two little maids nursed a rightly and a wrongly clothed and managed doll-baby, and explained matters with due gravity, while in another room two more children pieced the feet of stockings with the best parts of another pair, and the best scholars copied recipes for the guests. Does the British public still believe that "nothing useful" is taught in our schools? Jam made of carrot and beetroot was shown, and is said to be highly popular at a centre where school dinners are cooked. Glaxo tins found a new use, one placed on top of a small saucepan serving as a steamer. Potatoes were steamed in it at the same time as a little pudding in a jar, while soup cooked in the pot below.

